# Neural circuits underlying adaptation and learning in the perception of auditory space

**DOI:** 10.1016/j.neubiorev.2011.03.008

**Published:** 2011-11

**Authors:** Andrew J. King, Johannes C. Dahmen, Peter Keating, Nicholas D. Leach, Fernando R. Nodal, Victoria M. Bajo

**Affiliations:** Department of Physiology, Anatomy and Genetics, Sherrington Building, University of Oxford, Parks Road, Oxford OX1 3PT, UK

**Keywords:** Auditory cortex, Inferior colliculus, Sound localization, Binaural, Adaptive coding, Perceptual learning, Plasticity, Descending pathway, Corticocollicular projection, Chromophore-targeted laser photolysis, Cholinergic, Nucleus basalis

## Abstract

Sound localization mechanisms are particularly plastic during development, when the monaural and binaural acoustic cues that form the basis for spatial hearing change in value as the body grows. Recent studies have shown that the mature brain retains a surprising capacity to relearn to localize sound in the presence of substantially altered auditory spatial cues. In addition to the long-lasting changes that result from learning, behavioral and electrophysiological studies have demonstrated that auditory spatial processing can undergo rapid adjustments in response to changes in the statistics of recent stimulation, which help to maintain sensitivity over the range where most stimulus values occur. Through a combination of recording studies and methods for selectively manipulating the activity of specific neuronal populations, progress is now being made in identifying the cortical and subcortical circuits in the brain that are responsible for the dynamic coding of auditory spatial information.

## 1 Introduction

One of the principal functions of our sensory systems is to indicate the whereabouts of objects and events in the external environment. Although vision generally provides the most accurate spatial information, this is restricted to the visual field of the species in question. By contrast, the auditory system is able to register the presence of stimuli originating from any direction relative to the head. This omnidirectional function, and the capacity to localize sound sources even if they are at least partially occluded by other objects, confers considerable survival value and contributes to the ability of many species to find potential mates or prey or to avoid and escape from approaching predators. More generally, auditory spatial processing plays an important role in redirecting attention, and also helps listeners to pick out particular sources against a background of other sounds emanating from different directions in space (Schnupp et al., 2010).

Auditory localization relies on the physical separation of the ears on either side of the head. Thus, for sounds located to one side of the midline, the difference in path length to each ear produces an interaural difference in the time of sound arrival whose magnitude is determined by both the distance between the ears and angle subtended by the sound source relative to the head. An interaural difference in the level of the sound may also result from a combination of the spectral filtering effects produced by the external ears and acoustic shadow cast by the head. In mammals, binaural disparity cues are key to localization in the horizontal (or azimuthal) plane, with interaural time differences (ITDs) dominating at low frequencies and interaural level differences (ILDs) at high frequencies, while spectral cues provide the basis for vertical localization, for distinguishing between sources located in front of and behind the subject, and for the normally limited ability to localize sounds using one ear alone (King et al., 2001; Schnupp et al., 2010).

The ITD, ILD and spectral cue values corresponding to a given sound direction are determined by the physical dimensions of the head and external ears, which undergo pronounced changes during development and can also vary quite markedly between individuals within the same age band (Shaw and Teranishi, 1968; Xu and Middlebrooks, 1999; Schnupp et al., 2003; Campbell et al., 2008). Psychophysical studies have shown that humans can localize virtual acoustic space stimuli that simulate real sound sources more accurately when these headphone signals are based on acoustical measurements made from their own ears than from the ears of other subjects (Wenzel et al., 1993; Middlebrooks, 1999). This implies that experience of the cues provided by an individual's own head and ears shapes the functional organization of the brain circuits responsible for spatial hearing. While we might expect such experience-dependent plasticity to be greatest during development, particularly as the cues change in value as these structures grow, it is clear that the capacity for change persists into adulthood. Indeed, this dynamic processing of auditory localization cues seems to play a vital role in enabling listeners to interact effectively with their constantly changing acoustic environments, and provides the basis on which learning can improve their spatial abilities. Here, we review recent studies on the adaptive coding and plasticity of spatial hearing, which have started to reveal the neural circuits involved as well as the nature of the physiological changes that accompany shifts in perception.

## 2 Short-term adaptive coding

It is well established that the nervous systems of a range of species are adapted to the statistics of their sensory environment. Although some of the tuning of neural circuits to the regularities of the sensory environment has probably occurred over the course of evolution, much of this process seems to take place during development when the heightened plasticity of the nervous system provides the necessary flexibility for sensory experience to exert its effects (Blakemore and Cooper, 1970; Van der Loos and Woolsey, 1973; Zhang et al., 2001). Importantly, the resulting neural representations of the sensory environment appear to minimize redundancy and, thereby, maximize the efficiency with which the encountered signals are processed (Barlow, 1972). To further optimize information processing, sensory systems are also capable of adjusting their coding strategies over much shorter timescales, which allows them to take account of the often considerable fluctuations in input statistics between different sensory scenes.

Most of the evidence for such flexibility in sensory coding can be seen in the way the mature visual system adjusts to changes in the statistics of light intensity fluctuations (Baccus and Meister, 2002; Chander and Chichilnisky, 2001; Dunn and Rieke, 2006; Mante et al., 2005; Smirnakis et al., 1997) or of other visual stimulus dimensions (Brenner et al., 2000; Fairhall et al., 2001). Similarly, electrophysiological studies have shown that the responses of somatosensory (Garcia-Lazaro et al., 2007; Maravall et al., 2007) and auditory (Dean et al., 2005; Kvale and Schreiner, 2004; Nagel and Doupe, 2006; Wen et al., 2009) neurons at different processing levels can change with the composition of their input, such that the most frequently encountered stimuli are encoded most precisely.

Given this evidence from other stimulus dimensions, it would be surprising if the neural processing of auditory space was not optimized in a similar fashion. Indeed, previous work has shown that the sensitivity of neurons in the auditory cortex to interaural phase differences (Malone et al., 2002) and to virtual sound locations (Jenison et al., 2001) depends on the recent history of stimulation. However, it needs to be borne in mind that there are some fundamental differences between the processing of auditory space and, say, light intensity. For the latter, the processing of absolute stimulus values conveys little value – the detection of luminance *differences* across the visual scene is much more important than an accurate representation of a signal's absolute luminance. However, for auditory spatial processing, maintaining a stable representation of the *absolute* stimulus value, i.e. sound-source position, would seem to be more important than accurately registering spatial separations between stimuli.

**Fig. 1:**
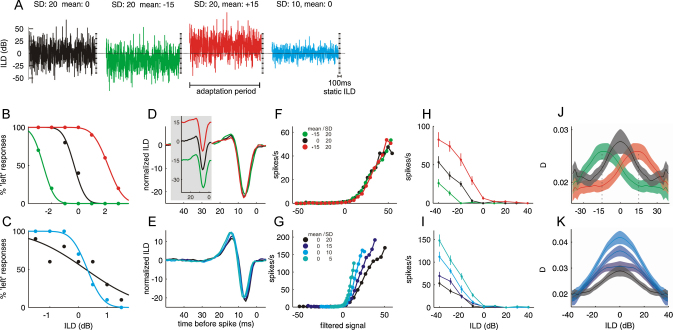
Auditory spatial processing adapts to stimulus statistics. (A) Human listeners and anesthetized ferrets were presented with noise sequences in which interaural level differences (ILD) rapidly fluctuated according to a Gaussian distribution. Negative values indicate that the sound level was higher in the contralateral ear (left ear for psychophysics). (B) Changing the mean of the distribution biased the perceived laterality of a subsequent stimulus, resulting in shifts in the listeners’ psychometric functions, which plot the percentage of trials that a subject perceived the sound presented over headphones to come from the left as a function of the ILD. (C) Changing the distribution's variance altered the listeners’ spatial sensitivity, as shown by increases (low variance) or decreases (high variance) in the slopes of the psychometric functions. (D–K) The responses of neurons in the inferior colliculus changed in line with these perceptual phenomena. For each neuron and each stimulus condition, spatial response properties were characterized both in terms of the components of a linear–nonlinear model (D–G) and a more conventional rate–ILD function (H, I). Slope and response variability of all rate-ILD functions were also analyzed further to obtain a population measure of neural sensitivity, the standard separation *D* (J, K). The linear–nonlinear model analysis describes neural coding using a two-stage process, consisting of linear filtering (D, E) of the stimulus by the neuron, which provides an estimate of the stimulus feature that best drives it, followed by spike generation according to a nonlinear function (F, G) of the similarity of the stimulus to that feature. This analysis revealed that neurons match their stimulus preference to the stimulus distribution's mean (inset in panel D shows filters before mean-subtraction), but retain similar gain (F) across different means. This results in large shifts in rate-ILD functions (H) and allows the population to maintain the highest sensitivity near the mean of the distribution (J). Across distributions with different variances, neurons largely retained their filter shape (E), but increased their gain as the variance was reduced (G). This resulted in steeper rate-ILD functions (I) and higher neural sensitivity in a low variance context (K).

Adapted from Dahmen et al. (2010).

Recent evidence provides some insight into how the processing of auditory space is affected by spatial input statistics (Fig. 1). By presenting human listeners over headphones with broadband noise sequences whose ILDs fluctuated rapidly according to a Gaussian distribution, and altering the mean or variance of that distribution (Fig. 1A), Dahmen et al. (2010) showed that the perception of auditory space strongly depends on the statistics of the sensory context. When the mean of the ILD distribution was changed, the perceived laterality of a subsequent stimulus was shifted away from the mean (Fig. 1B). Manipulating the variance of the stimulus distribution also affected perception, such that spatial sensitivity improved as the variance was decreased and declined when the variance was increased (Fig. 1C).

Dahmen et al. (2010) also looked for a possible neural substrate for these perceptual changes by presenting essentially the same stimuli to neurons recorded in the inferior colliculus (IC) of anesthetized ferrets. They found that the neurons change their response properties in a way that is highly consistent with the perceptual phenomena. As a result of adaptation to the mean, IC neurons can respond almost identically to very different ILD values, so long as those stimuli lie at the same distance to the mean of the ILD distribution within which they are presented (Fig. 1D, F, H, J). Thus, in the rate–ILD plot shown in Fig. 1H, the neuron produced the same response to ILDs of −30, −15 and −3 dB following adaptation to stimulus distributions with mean ILDs of −15, 0 and +15 dB, respectively. Relying on these neurons for an estimate of the absolute ILD of a signal will therefore result in precisely the type of mean-biased judgements seen psychophysically. IC neurons adjust their gain if the variance of the ILD distribution changes: if the variance goes down, they represent the same difference in input with a larger difference in firing rate (Fig. 1E, G, I, K). Again, the nature of this neural adaptation is consistent with the observed relationship between perceptual sensitivity and stimulus variance.

Further electrophysiological evidence from the same study (Dahmen et al., 2010), as well as other psychophysical results (Getzmann, 2004; Kashino, 1998; Maier et al., 2010; Sach et al., 2000), suggest that an inability to make mean-independent judgements of absolute ILD values may be the cost for the brain's attempt to maintain the highest perceptual sensitivity in that region of space where the majority of stimuli occur. A cost may also be associated with variance adaptation because the observed increase in gain with shrinking variance might produce distortions in the perception of auditory space that, at the same time as improving sensitivity to changes in ILD, result in a systematic overshooting of absolute location judgements. Given the aforementioned importance of creating and maintaining accurate neural representations of sound-source location, which is supported by the studies of learning-induced plasticity that we review in the following sections, this apparent emphasis on relative spatial differences is surprising and requires further investigation.

The IC receives converging inputs from each of the brainstem nuclei that process the different cues to sound-source location (Loftus et al., 2010), and represents the first stage at which neurons exhibit sensitivity to ILDs, ITDs and spectral cues (Chase and Young, 2008). The ITD discrimination thresholds measured for IC neurons in anesthetized guinea pigs are comparable to those measured psychophysically in humans (Shackleton et al., 2003). Similarly, Dahmen et al. (2010) observed a close correspondence in the way that ferret IC neurons and human listeners adjust their ILD sensitivity to stimulus statistics. Together with other evidence for adaptation to stimulus statistics at very early levels of the sensory processing hierarchy (Baccus and Meister, 2002; Chander and Chichilnisky, 2001; Dunn and Rieke, 2006; Smirnakis et al., 1997; Wen et al., 2009), these results suggest that higher-level, task-dependent effects play little or no role in the short-term adaptive processing of auditory spatial information. This does not, however, rule out the possibility that corticofugal modulation of midbrain neurons may also be involved, which, as we discuss below, can have a profound effect on auditory spatial processing (Bajo et al., 2010; Nakamoto et al., 2008).

## 3 Adaptation to altered spatial cues during development

In addition to adjustments in neural processing that result from changes in the statistics of recent sensory experience, longer-lasting changes in inputs can induce plasticity in the brain's representation of auditory spatial cues. As we have already pointed out, the dependence of these cues on the dimensions of the head and ears suggests that accurate auditory localization is a process that has to be learned by experience of the cues available to each individual. Consequently, experimentally manipulating these cues during development can have a profound effect on the spatial response properties of auditory neurons and on the way in which those cues are perceived.

An effective, and potentially reversible, way of altering the spatial cue values corresponding to each direction in space is to introduce a unilateral conductive hearing loss. This has been shown to affect sound localization performance in different ways depending on the precise nature, timing and duration of hearing loss, as well as the species in which it occurs. Localization behavior in barn owls, for example, readily adapts if one ear is plugged early in development (Knudsen et al., 1984a), with subsequent restoration of normal hearing initially leading to large errors that gradually disappear over time (Knudsen et al., 1984b). In general, however, barn owls perform best using localization cues that they have experienced during the first eight weeks of life, suggesting that this period may be particularly important for the development of veridical sound localization (Knudsen et al., 1984a,b).

At a neural level, earplug adaptation in barn owls is paralleled by compensatory shifts in the ITD and ILD tuning of cells in the optic tectum (Mogdans and Knudsen, 1992) and external nucleus of the inferior colliculus (Mogdans and Knudsen, 1993), both of which contain a topographic map of space in these animals. Adaptive shifts in neural tuning have also been observed in these midbrain nuclei (Gold and Knudsen, 2000a,b) and in both the thalamus (Miller and Knudsen, 2003) and forebrain (Miller and Knudsen, 2001) of barn owls following unilateral implantation of a device that filters auditory input in a manner broadly comparable with that of an earplug. Because these shifts are absent at the level of the central nucleus of the IC, but emerge at subsequent levels of processing, it is thought that plasticity in binaural sensitivity is implemented by changing the patterns of connectivity between the central and external nuclei of the IC (Gold and Knudsen, 2001). This rewiring of midbrain connections appears to enable the barn owl to adapt to unilateral hearing loss by learning abnormal frequency-specific mappings between individual cues and specific spatial positions, thereby maintaining an accurate representation of space.

**Fig. 2:**
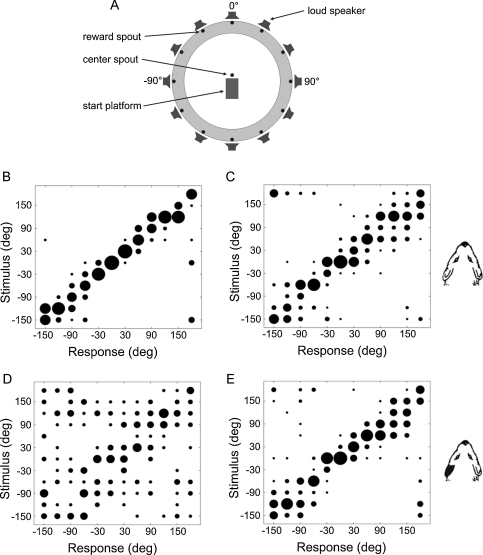
Effects of chronic monaural occlusion in infancy on the accuracy and precision of auditory localization. (A) Schematic view of the chamber used to measure the sound localization ability of ferrets. Animals were trained to stand on a start platform and initiate a trial by licking the center spout. Each trial consisted of a Gaussian noise burst (0–22 kHz, 200 ms duration) presented quasirandomly from one of 12 speakers placed at 30° intervals in the azimuthal plane. Amplitude spectra were divided up into 1/6th octave bands and the level of each band was varied independently, with all variations in level chosen randomly on each trial from a normal distribution with a standard deviation of 5 dB. Within each testing session, five sound levels ranging randomly from 56 to 84 dB SPL were used to minimize loudness cues. Ferrets were rewarded for approaching and licking the spout associated with the speaker that had been triggered. (B–E) Stimulus–response plots showing sound localization performance in ferrets. Each plot illustrates the distribution of responses as a function of stimulus location, with the size of each dot determined by the proportion of responses made to different spout locations. Data are plotted for normally reared ferrets in the absence of an earplug (B), ferrets reared with an earplug immediately after the earplug was removed (C), normally reared ferrets following the insertion of an earplug for the first time (D), and ferrets reared with an earplug with the earplug still in place (E). Note that the animals reared with one ear occluded could localize sound almost as well as the normally reared controls.

Although the mechanisms underlying the particularly accurate sound localization abilities of the barn owl show a number of specializations that have not been observed in other species, studies in mammals have confirmed the adaptive nature of sound localization during development. In particular, adult ferrets tend to exhibit largely normal sound localization behavior after being raised with a unilateral earplug (King et al., 2000; Fig. 2). The after-effects associated with earplug removal, however, are much less pronounced than those reported in barn owls, suggesting that the mechanisms underlying adaptation may differ across species.

Electrophysiological recordings in the ferret superior colliculus (SC) have shown that – as in the optic tectum, its avian homologue – auditory spatial tuning adjusts to the presence of a plug in one ear during development, so that a near normal map of auditory space emerges (King et al., 1988, 2000). But in contrast to the barn owl, this adaptive plasticity does not seem to be based on a retuning of the neurons to the altered binaural cues. Thus, the auditory spatial preferences of ferret SC neurons remain remarkably similar irrespective of whether an earplug is present or not, the most notable change being a slight coarsening of the map of space when the earplug is in place (King et al., 2000). The most parsimonious interpretation of both the behavioral and electrophysiological data from ferrets raised with a unilateral conductive hearing loss is that the development of near-normal sound localization may involve a reweighting of different spatial cues, so that less dependence is placed on the binaural cues, especially the ILDs, that are most affected by the earplug.

Testing this possibility requires measuring neuronal sensitivity to different auditory spatial cues. Cats reared with unilateral atresia of the external ear canal – the effects of which are broadly similar to those of an earplug – fail to show changes in ITD tuning in the primary auditory cortex (A1), though shifts in ILD tuning were observed (Brugge et al., 1985). The direction of those shifts, however, was opposite to that found in barn owls, and therefore inconsistent with adaptation to the attenuated input in one ear. Brugge et al. (1985) attributed these changes in ILD sensitivity to weakened inhibitory input resulting from residual hearing loss in the atretic ear. A rather different result was reported in cat IC, with few neurons exhibiting sensitivity to ILDs following the restoration of a balanced binaural input, which appeared to be due to a reduced inhibitory input from the intact ear (Moore and Irvine, 1981).

Electrophysiological recordings in rats have, however, provided more consistent evidence for central auditory system plasticity. Thus, unilateral ear canal ligation results in a weakening of inhibitory input from the manipulated ear at the level of the IC (Silverman and Clopton, 1977), effects that become smaller as the age of onset of hearing loss is delayed (Clopton and Silverman, 1977). More recent work has confirmed both of these findings, and suggests that similar changes may occur in A1, albeit in a more pronounced way and with a different dependence on the age of onset of hearing loss (Popescu and Polley, 2010). These results are therefore consistent with those reported for cat A1, and suggest that inputs from the formerly occluded ear may be progressively weakened at higher levels of the neuroaxis as competitive plasticity increasingly favors the representation of the unaffected ear.

Taken together, these data suggest that mammals may not be able to learn abnormal mappings between binaural spatial cues and specific spatial positions during development, and may instead learn to ignore the input provided by the less effective ear. In theory, this could lead to profoundly impaired sound localization abilities, but, so far, this has been tested only in ferrets raised with an earplug in one ear. Because those animals do learn to localize sounds accurately and develop a map of space in the SC, the auditory system's response to these abnormal inputs is also likely to include learning to rely more on the unaltered spatial information provided by spectral cues in the unaffected ear.

## 4 Adaptation to altered spatial cues in adulthood

Once the head and ears attain their adult size, thereby stabilizing the values of the auditory spatial cues, it might be expected that the potential for plasticity in the neural circuits responsible for spatial hearing would decline. This indeed seems to be the case as several studies have reported that, compared to the changes reported after altering auditory experience during infancy, exposure to abnormal spatial cues produced by a unilateral conductive hearing loss in adults is much less effective in inducing adaptive changes in auditory localization (Knudsen et al., 1984a,b; King et al., 2000).

While these findings point to the existence of a sensitive period of development within which neural circuits can be shaped by experience, behavioral studies in humans (Bauer et al., 1966; Hofman et al., 1998; Van Wanrooij and Van Opstal, 2005; Kumpik et al., 2010) and ferrets (Kacelnik et al., 2006) have demonstrated that the mature auditory system is, in fact, capable of adapting to markedly altered spatial cue values. Our own experiments in ferrets indicate that the key to this ability is the behavioral context (Kacelnik et al., 2006). Thus, adult animals with unilateral earplugs remain severely impaired in their ability to localize sound for at least several weeks if they receive no behavioral training during this time. By contrast, if the animals are trained on a sound localization task, adaptive changes take place within a few days, and the extent and rate at which their localization judgments improve is determined by the frequency of training.

In terms of the possible neural origins of such plasticity, experiments in barn owls once again point to the involvement of the auditory space map in the optic tectum, which exhibits much greater experience-dependent plasticity in adult birds that are allowed to hunt with live prey than those fed on dead mice (Bergan et al., 2005). In mammals, however, attention has focused principally on the auditory cortex, rather than on the midbrain. There are two reasons for this. First, the behavioral deficits observed followed aspiration lesions (Heffner and Heffner, 1990; Jenkins and Masterton, 1982; Kavanagh and Kelly, 1987; Nodal et al., 2010) or reversible inactivation (Malhotra et al., 2008; Smith et al., 2004) of the auditory cortex have highlighted its critical role in normal sound localization. Second, various types of auditory perceptual learning are associated with changes in the response properties of auditory cortical neurons (Dahmen and King, 2007).

**Fig. 3:**
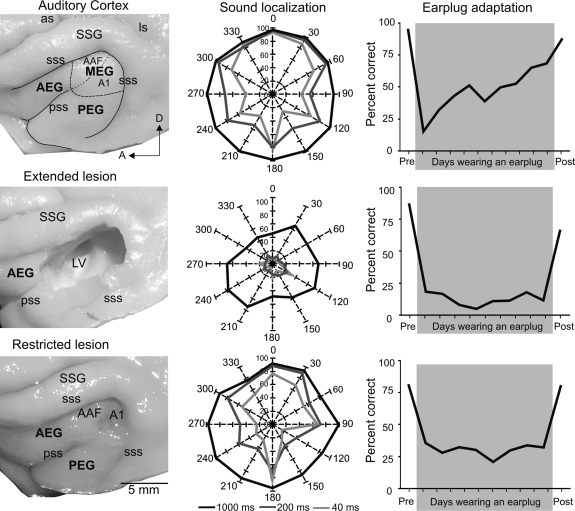
Lesions of auditory cortex impair sound localization and training-induced plasticity. Left column shows the extent of the auditory cortical lesions in different animals. The top panel shows the main subdivisions superimposed over the auditory cortex on a ferret brain. The middle panel shows an extended lesion that comprised the whole cortical thickness, including the white matter and most of the auditory cortex. The bottom panel shows a restricted lesion affecting only the primary auditory cortex (A1) while preserving the underlying white matter. The plots in the middle column show the percentage of correct responses in a 12-speaker approach-to-target task (see Fig. 2A) at 3 different sound durations (1000, 200 and 40 ms). When short duration sounds were used, control ferrets exhibited reduced spatial accuracy at lateral and posterior positions compared with anterior positions (top panel). A1 lesions degraded the accuracy with which brief sounds were localized, without affecting performance at longer durations (bottom panel), whereas larger deficits, affecting performance at all sound durations tested, were observed following extensive lesions of the auditory cortex (middle panel). The right column shows the ability of the animals to adapt to the altered spatial cues produced by plugging one ear. The top panel shows data from a control ferret: after an initial fall in the percentage of correct scores following earplug insertion, the animal's performance gradually recovered with training to almost reach pre-plug levels. This training-induced plasticity depends on the integrity of the auditory cortex as no improvement in performance was observed in animals with cortical lesions, even if the region aspirated was restricted to A1 (middle and bottom panels). *Abbreviations*: as, ansinate sulcus; A1, primary auditory cortex; AAF, anterior auditory field; AEG, anterior ectosylvian gyrus; ls, lateral sulcus; LV, lateral ventricle; MEG, middle ectosylvian gyrus; PEG, posterior ectosylvian gyrus; pss, pseudosylvian sulcus; SSG, suprasylvian gyrus; sss, suprasylvian sulcus.

Although a reduced ability to localize sound results when A1 alone is silenced, larger deficits are observed when surrounding auditory cortical areas are affected as well (Fig. 3), suggesting that different parts of the auditory cortex are required for the processing of spatial information. Indeed, there is both physiological (Harrington et al., 2008; Miller and Recanzone, 2009; Bizley and King, 2011) and behavioral (Malhotra et al., 2008) evidence that certain non-primary areas make larger contributions to sound localization. Exactly how the cortex underpins the perception of auditory space remains uncertain, but the failure to find a topographic representation equivalent to that present in the SC (Palmer and King, 1982) has prompted the idea that sound-source location is encoded by the spatial distribution of activity across populations of cortical neurons (Miller and Recanzone, 2009; Stecker et al., 2005).

Lesion studies have highlighted the involvement of the auditory cortex not only in sound localization under normal hearing conditions, but also in the ability of animals to relearn to localize sound when spatial cues are disrupted by temporarily occluding one ear. Thus, the training-induced recovery of accurate sound localization found in ferrets with unilateral earplugs is absent in animals in which the auditory cortex had previously been lesioned bilaterally (Nodal et al., 2010). The loss of this adaptive plasticity was found both in animals in which extensive lesions that included a substantial part of the auditory cortex had been made and in those in which the lesions were restricted to A1. Importantly, these learning deficits were not due to an impaired ability to localize the sounds, as no learning was observed even for long duration sounds that could still be localized normally in the absence of the earplug (Fig. 3).

These experiments show that presenting animals with the more challenging situation of having to compensate for changes in the cues that are available to localize sound can reveal a greater involvement of A1 than is apparent from simply measuring localization performance with normal cue values. Given the distributed nature of spatial processing in the auditory cortex, it is perhaps not surprising that other cortical areas also seem to play a part in learning-induced plasticity (Nodal et al., 2008). Thus, exploring the relative contribution of these areas to task-dependent plasticity and learning has the potential to provide valuable insights into how behaviorally relevant information is represented across the auditory cortex.

**Fig. 4:**
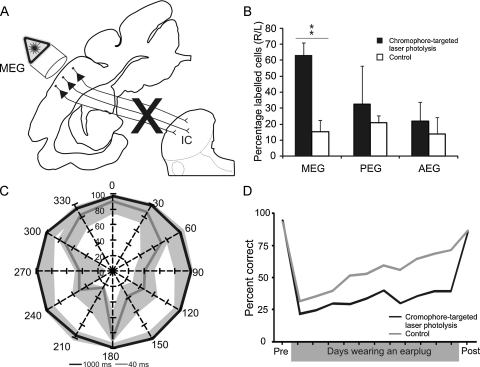
The auditory corticocollicular projection is required for training-induced plasticity of spatial hearing. (A) Layer V corticocollicular pyramidal cells were retrogradely labeled by making injections into the inferior colliculus of fluorescent microspheres coated with a specific chromophore. Apoptosis was selectively triggered in the labeled neurons by applying near-infrared light (*λ* = 670 nm) to the primary auditory cortex in the middle ectosylvian gyrus. (B) The loss of corticocollicular neurons in the left auditory cortex is indicated by expressing the number of labeled neurons on that side as a percentage of those in the right hemisphere, contralateral to the injection sites in the inferior colliculus. This ipsilateral/contralateral ratio is about 15–20% in control cases, but much larger following chromophore-targeted laser photolysis, indicating a substantial loss of labeled cells in the targeted auditory cortex (**P < 0.01). (C) Sound localization accuracy in the horizontal plane was unchanged even when very brief sound durations were used, with no differences between the ferrets with corticocollicular lesions and controls. The lines depict the mean values for the lesioned cases at two different sound durations, 1000 ms in black and 40 ms in gray, and the gray bands correspond to 1 s.d. on either side of the mean values achieved by control ferrets. (D) The ability to adapt to altered spatial cues caused by the monaural insertion of an earplug was impaired in ferrets with corticocollicular lesions (black), with no improvement observed with training compared with control cases (gray). The lines are mean values for each group. *Abbreviations*: AEG, anterior ectosylvian gyrus; IC, inferior colliculus; MEG, middle ectosylvian gyrus; PEG, posterior ectosylvian gyrus.

Another issue concerning the role of different auditory cortical fields in sound localization and its recalibration by experience is the involvement of neurons located in different cortical layers. A recent study showed that auditory spatial learning is critically dependent on the descending projection from A1 to the auditory midbrain (Bajo et al., 2010; Fig. 4). To demonstrate this, cortical layer V pyramidal cells were first retrogradely labeled by injecting fluorescent microbeads conjugated with chlorine e_6_ in the left IC, and subsequently killed by illuminating the ipsilateral auditory cortex with near-infrared light (Fig. 4A). The ratio of crossed to uncrossed corticocollicular projection neurons is normally about 20%, and on the basis of the threefold increase in this ratio, Bajo et al. (2010) estimated that chromophore-targeted laser photolysis removed about two thirds of the A1 neurons that project to the IC, without affecting those in surrounding cortical areas (Fig. 4B). No change in sound localization accuracy was observed, even at short stimulus sound durations (Fig. 4C), indicating that loss of the majority of layer V corticocollicular neurons does not result in the same localization deficits that are produced by complete aspiration lesions of A1. However, the spatial plasticity that normally occurs after altering the interaural balance by plugging one ear was severely impaired (Fig. 4D), suggesting that corticofugal pathways are essential for recalibration of the brain's representation of auditory space.

What information the auditory cortex provides to IC neurons via these descending projections to allow auditory spatial learning to take place is not yet known. Electrical stimulation or inactivation of cortical neurons can, however, modify almost every aspect of the response properties of IC neurons, including their sensitivity to sound frequency, intensity and location (Luo et al., 2008; Ma and Suga, 2001; Nakamoto et al., 2008; Suga, 2008; Zhou and Jen, 2005). The shifts in ILD sensitivity of IC neurons that occur following cortical cooling (Nakamoto et al., 2008) or, as we saw above, in response to the recent stimulus statistics (Fig. 1; Dahmen et al., 2010) highlight the dynamic nature of spatial processing in the IC. But as with monaural occlusion during infancy, adaptation to a unilateral hearing loss in adults seems to involve learning to ignore the abnormal cue values while placing more emphasis on the cues that are less affected by the earplug (Kacelnik et al., 2006; Kumpik et al., 2010). Thus, future studies will need to address how the processing of different spatial cues, including ITDs and spectral cues, changes as learning occurs.

While recent work has focussed on the influence of descending inputs from the auditory cortex on spatial coding in the midbrain, there is also growing evidence for experience-dependent plasticity at lower levels of the auditory brainstem, including in the circuits involved in the initial processing of sound localization cues (Tzounopoulos and Kraus, 2009). In fact, behavioral adaptation by adult ferrets to a unilateral earplug is impaired following midline lesions of the olivocochlear bundle (Irving et al., 2011), which originates in the superior olivary complex where sensitivity to binaural cues is first derived. In principle, activation of olivocochlear efferents could produce a frequency-specific adjustment in the output from the cochlea in one or both ears, thereby altering the localization cue sensitivity of neurons at higher levels of the auditory pathway.

## 5 Top-down signals and auditory plasticity

We have so far considered the role of different levels of the auditory pathway, and the connections between them, in adaptive coding and plasticity. An important aspect of auditory processing, however, especially during learning-induced plasticity, is the possible role that attention might exert to provide meaning to the sensory stimulus. The spectrotemporal receptive fields (STRF) of ferret A1 neurons exhibit rapid task-dependent plasticity when the animals carry out behavioral tasks that require them to pick out a target sound against a background of reference sounds (Fritz et al., 2007). Because STRF plasticity is seen only when the ferrets perform tasks that require them to attend to these sounds, and not during passive presentation of the same stimuli, it seems likely that top-down inputs to the auditory cortex are responsible for triggering these changes. A likely source of these top-down effects is the frontal cortex, where neurons often respond to the target sounds only during behavior (Fritz et al., 2010). Interestingly, the coherence between local field potentials recorded simultaneously in the frontal and auditory cortices changes during task execution, suggesting that connections between these brain regions might provide the attentional control needed to induce STRF plasticity in A1.

The likely importance of top-down effects in auditory learning has also been demonstrated. Thus, Polley et al. (2006) found that the response properties of neurons in both A1 and a secondary cortical area show a task specific reorganization in rats trained to recognize particular sound frequencies or sound intensities. Because the same set of sounds was used in each case, this task-dependent plasticity implies that the sensitivity of individual neurons can change in different ways to enhance specific aspects of perception. Indeed, although passive exposure to particular stimuli can change the response properties of cortical neurons in mature animals (Pienkowski and Eggermont, 2009), it seems to be the dependence on the behavioral salience of the stimuli that sets adult learning apart from the plasticity that is observed during development.

If a particular tone frequency acquires behavioral significance by combining it with a mild electric shock, the best frequencies of cortical neurons shift toward that value (Bakin and Weinberger, 1990). These modulations in cortical tuning are rapid in onset and relatively long-lasting, even persisting for several weeks given sufficient training (Weinberger et al., 1993). Subsequent studies have shown that auditory fear conditioning produces experience-dependent plasticity not only in the cortex, but also sub-cortically in both the thalamus (Edeline and Weinberger, 1991) and IC (Gao and Suga, 2000).

Behavioral salience can be simulated *in vivo* by electrical microstimulation of sources of cortical modulatory input. In particular, stimulation of the nucleus basalis (NB), the region of the cholinergic basal forebrain that projects to the neocortex, results in the release of acetylcholine (Casamenti et al., 1986; Rasmusson et al., 1992), which, in turn, facilitates auditory thalamocortical synaptic transmission and increases cortical excitability (Metherate and Ashe, 1993). Pairing electrical stimulation of the NB with sound presentation induces stimulus-specific changes in cortical receptive fields that closely resemble those seen after behavioral training (Bakin and Weinberger, 1996; Yan and Zhang, 2005). Thus, neuronal best frequencies are shifted towards those of the paired stimuli (Bakin and Weinberger, 1996; Ma and Suga, 2003, 2005), the representations of which are increased in primary and secondary auditory fields (Kilgard and Merzenich, 1998; Puckett et al., 2007). This pairing paradigm also induces similar shifts in the best frequencies of IC neurons, which are dependent on activity in the auditory cortex (Zhang et al., 2005), providing further evidence for the role of corticofugal descending connections in experience-dependent plasticity.

Blockade of cortical acetylcholine receptors prevents the receptive field plasticity produced by both auditory fear conditioning (Ji and Suga, 2003) and NB stimulation (Miasnikov et al., 2001; Yan and Zhang, 2005; Zhang et al., 2005). This is also the case if the projection to cortex is specifically lesioned by injecting a targeted cholinergic neurotoxin into the NB (Kilgard and Merzenich, 1998). However, although acetylcholine-dependent NB stimulation leads to the formation of specific associative memory for the paired tone frequency (Miasnikov et al., 2008), behavioral evidence for the dependence of learning-induced auditory plasticity on the cholinergic basal forebrain is lacking.

**Fig. 5:**
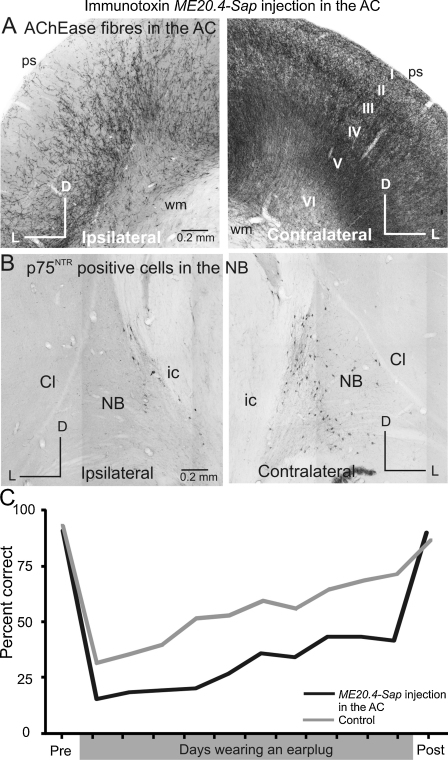
Loss of cholinergic input from the nucleus basalis to the auditory cortex appears to disrupt learning-induced plasticity. The number of AChE*ase* fibers in the auditory cortex (A) and of the low affinity neurotrophin receptor (p75^NTR^) positive cells in the nucleus basalis (B) was significantly lower following injections of the immunotoxin ME20.4-SAP in the ipsilateral auditory cortex. ME20.4-SAP comprises a monoclonal antibody specific for the p75^NTR^ membrane-bound receptor, conjugated to saporin, a ribosome-inactivating enzyme. Once bound to the external cell membrane, the saporin toxin is internalized and prevents protein synthesis, resulting in neuronal cell death. The p75^NTR^ receptor is primarily expressed by the cholinergic cells of the basal forebrain and, after being injected into the auditory cortex, ME20.4-SAP is taken up only by cortical cholinergic afferents. (C) The ability to adapt to altered spatial cues caused by the monaural insertion of an earplug was reduced in ferrets in which cholinergic innervation to the cortex had been compromised by this method. *Abbreviations*: I to VI, layers of the cortex; Cl, claustrum; D, dorsal; ic, internal capsule; L, lateral; NB, nucleus basalis; ps, pial surface; wm, white matter.

Recent work in ferrets has investigated the involvement of cholinergic neuromodulation in the ability of adult ferrets to localize sounds in space and to adapt to the altered spatial cues produced by plugging one ear (Leach et al., 2011). Fig. 5 shows a single case with multiple injections of a specific neurotoxin throughout the left auditory cortex, from where it is transported retrogradely back to the NB and induces cell death. This produced a substantial decrease (of around 70%) in both the number of cholinergic (p75^NTR^ immunopositive) cells in the ipsilateral NB and acetylcholinesterase-positive fibers in A1 on that side of the brain (Fig. 5A and B). Behaviorally, these cholinergic lesions produced a modest impairment in the localization of brief sounds (40–100 ms in duration), as shown by the lower percentage of correct scores and higher error rates compared with control animals. Interestingly, no differences in performance were observed between the left and right hemifields, as might be anticipated from unilateral cortical lesions, a result which might indicate a more global role for acetylcholine. Indeed, McGaughy and colleagues have shown that impairments in cortical cholinergic transmission produce substantial deficits in sustained attention and stimulus detection thresholds during a visual discrimination task (McGaughy et al., 1996; McGaughy and Sarter, 1998). The capacity of the ferrets to relearn to localize sound accurately in the presence of a unilateral hearing loss was also slightly reduced compared to controls (Fig. 5C), indicating a likely role for the cholinergic system in auditory spatial learning.

## 6 Conclusions

The studies reviewed in this article highlight the highly flexible nature of auditory spatial coding. From recalibrating the tuning properties of auditory neurons according to the context in which sounds occur to the longer-lasting plasticity that allows individuals to utilize the cues provided by their own ears to localize sounds accurately, experience is used over multiple time scales to continually reshape the representation of sensory signals in the brain. This adaptive processing is presumably critical for the perception of real-world sounds, which often occur against a dynamic and complex background of other sensory cues, and must underlie the brain's capacity to adjust to the abnormal inputs that result from hearing loss and its restoration.

Although progress has been made in identifying key elements in the neural circuitry that are responsible for adaptive spatial coding and plasticity, we still have only a rudimentary understanding of how different levels of processing, such as the auditory cortex and midbrain, work together to give rise to changes in auditory perceptual abilities or how neural coding strategies within those circuits are shaped by experience. It also remains to be seen whether neural coding adaptation that is driven by the statistics of the environment is influenced by top-down inputs and perceptual learning. Future behavioral and neurophysiological studies in this area will need to focus on how different spatial cues are combined to form a coherent representation of space, and will undoubtedly benefit from the application of the growing number of methods that are becoming available for manipulating activity in specific neural circuits.
